# Cloning and Expression Analysis of cDNAs Encoding ABA 8'-Hydroxylase in Peanut Plants in Response to Osmotic Stress

**DOI:** 10.1371/journal.pone.0097025

**Published:** 2014-05-13

**Authors:** Shuai Liu, Yan Lv, Xiao-Rong Wan, Li-Mei Li, Bo Hu, Ling Li

**Affiliations:** 1 Guangdong Provincial Key Lab of Biotechnology for Plant Development, College of Life Sciences, South China Normal University, Guangzhou, China; 2 College of Life Sciences, Zhongkai University of Agriculture and Engineering, Guangzhou, China; Key Laboratory of Horticultural Plant Biology (MOE), China

## Abstract

Abscisic acid (ABA) catabolism is one of the determinants of endogenous ABA levels affecting numerous aspects of plant growth and abiotic-stress responses. The major ABA catabolic pathway is triggered by ABA 8'-hydroxylation catalysed by ABA 8'-hydroxylase, the cytochrome P450 CYP707A family. In this study, the full-length cDNAs of AhCYP707A1 and AhCYP707A2 were cloned and characterized from peanut. Expression analyses showed that *AhCYP707A1* and *AhCYP707A2* were expressed ubiquitously in peanut roots, stems, and leaves with different transcript accumulation levels, including the higher expression of *AhCYP707A1* in roots. The expression of *AhCYP707A2* was significantly up-regulated by 20% PEG6000 or 250 mmol/L NaCl in peanut roots, stems, and leaves, whereas the up-regulation of *AhCYP707A1* transcript level by PEG6000 or NaCl was observed only in roots instead of leaves and stems. Due to the osmotic and ionic stresses of high concentration of NaCl to plants simultaneously, low concentration of LiCl (30 mmol/L, at which concentration osmotic status of cells is not seriously affected, the toxicity of Li^+^ being higher than that of Na^+^) was used to examine whether the effect of NaCl might be related to osmotic or ionic stress. The results revealed visually the susceptibility to osmotic stress and the resistance to salt ions in peanut seedlings. The significant up-regulation of *AhCYP707A1*, *AhCYP707A2* and *AhNCED1* transcripts and endogenous ABA levels by PEG6000 or NaCl instead of LiCl, showed that the osmotic stress instead of ionic stress affected the expression of those genes and the biosynthesis of ABA in peanut. The functional expression of *AhCYP707A1* cDNA in yeast showed that the microsomal fractions prepared from yeast cell expressing recombinant AhCYP707A1 protein exhibited the catalytic activity of ABA 8'-hydroxylase. These results demonstrate that the expressions of *AhCYP707A1* and *AhCYP707A2* play an important role in ABA catabolism in peanut, particularly in response to osmotic stress.

## Introduction

The plant hormone abscisic acid (ABA) regulates many important physiological and developmental processes in plants as well as adaptive responses to environmental stresses [1]. In theory, endogenous ABA content should be maintained by a balance between biosynthetic and catabolic activities. Thus, to further understand the molecular mechanism that controls the ABA contents in plants, the genes and enzymes in biosynthesis and catabolism of ABA must be investigated in detail. A number of enzymes for ABA biosynthesis have been identified by various genetic or biochemical approaches [2]. One of these, the 9-*cis*-epoxycarotenoid dioxygenase (NCED), cleaves 11,12 double bonds of C_40_ carotenoids and produces the C_15_ precursor of ABA. This step is thought to be a critical reaction for *de novo* ABA biosynthesis in plants [3]–[5]. There are five members of the NCED family in the Arabidopsis genome. As the NCED family members exhibit various tissue specificities and expression patterns, it is suggested that each plays a distinct role [6]. Drought stress induced *AtNCED3* predominantly among Arabidopsis *NCED* genes, therefore AtNCED3 is regarded as the most important enzyme for drought-inducible ABA biosynthesis [7]. We have characterized a peanut *NCED* gene, *AhNCED1*, and demonstrated that the expression of *AhNCED1* gene plays an important role in the regulation of ABA level during water stress, and that water-stress tolerance of Arabidopsis plants can be improved by ectopic expression of the *AhNCED1* gene causing accumulation of endogenous ABA [8], [9].

Although much is known about ABA biosynthesis in plants, our knowledge about the catabolic pathway of ABA is still relatively limited [2]. ABA is catabolized into inactive forms by either oxidation or conjugation [2]. The oxidative pathways play a pivotal role in various physiological processes. The major oxidative pathway is triggered by the hydroxylation of C'-8 to form 8'-hydroxy ABA (8'OH-ABA), which is unstable and can be spontaneously isomerized to phaseic acid (PA), and finally reduced to dihydrophaseic acid (DPA) [2]. It has been predicted that ABA 8'-hydroxylase belongs to the cytochrome P450 (CYP) monooxygenase superfamily and is named as CYP707A [2], [10].

In recent years, considerable progress has been made in the identification and characterization of cDNAs encoding ABA 8'-hydroxylase, including four *CYP707A* genes in Arabidopsis [11], [12], two in barley [13], [14], two in rice [15], three in bean [16], five in maize [17], ten in soybean [18], and three in potato [19]. These investigations show that the expression of plant *CYP707A* genes is regulated developmentally and environmentally. In Arabidopsis, *CYP707A*s are induced by exogenous ABA treatment, dehydration and rehydration [11], [12]. The induction of *CYP707A*s is likely to be important for the maintenance of endogenous ABA levels, especially when plants have to inactivate ABA promptly after release from dehydration [20]. Umezawa et al [21] further showed that CYP707A3 plays a prominent role in ABA catabolism during the dehydration and rehydration processes of Arabidopsis plants. In bean, *PvCYP707A3* transcripts significantly increased in response to dehydration, no changes of mRNA levels of *PvCYP707A1* and *PvCYP707A2* were found, however, mRNA levels of *PvCYP707A1* and *PvCYP707A2* in dehydrated leaves rapidly increased in response to rehydration [16]. Transgenic *Nicotiana sylvestris* plants over-expressing *PvCYP707As* displayed a wilty phenotype with reduced ABA levels and increased PA levels, and it has been suggested that to increase ABA levels further by genetically repressing ABA 8'-hydroxylase would be a more promising strategy than overexpressing NCEDs [16], [22], [23].

Strong induction of *CYP707A1* and *CYP707A4* transcripts and moderate increase of *CYP707A2* and *CYP707A3* mRNA levels were observed in Arabidopsis subjected to 250 mmol/L NaCl stress [12]. The expressions of rice *OsCYP707A5* in leaves [15] and all soybean *GmCYP707A*s in roots [18] were also increased sharply by high salinity. Salt stress involves both osmotic and ionic effects [24]. We, therefore, attempted to study these two kinds of stress separately with the aid of low concentration of LiCl treatment [25].

Osmotic stress is one of the major abiotic stresses that limit the growth and production of plants. The mechanisms of osmotic stress response have been investigated most extensively in Arabidopsis [26], [27], whereas the biological functions of many genes related to osmotic stress response are still largely unknown in agricultural crops. Therefore, it is important to study the functions of stress-related genes to provide the basis for engineering greater stress tolerance in crops. We have used peanut, an economically important oil and protein rich crop, to investigate this subject [8], [9], [28]–[33]. In the present study, we report the cloning and characterization of two genes encoding ABA 8'-hydroxylase from peanut, designated as *AhCYP707A1* and *AhCYP707A2*, respectively. The heterologous expression of *AhCYP707A1* cDNA in yeast showed the catalytic activity of ABA 8'-hydroxylase of the recombinant AhCYP707A1 protein. Expressions of *AhCYP707A1* and *AhCYP707A2* genes were ubiquitous in peanut roots, stems and leaves with different transcript levels, and were regulated osmotically, as shown by responses to osmotic stress instead of ionic stress.

## Materials and Methods

### Peanut plants and growth conditions

Seeds of peanut (*Arachis hypogaea* L. cv Yueyou 7) were sown in pots with a potting mixture of vermiculite, perlite and soil (1∶1∶1), and grown in a growth chamber with 16 h of light from fluorescent and incandescent lamps (200 µmol m^−2^ s^−1^) followed by 8 h of darkness at 28°C. Plants were watered daily with half-strength Murashige and Skoog nutrient solution [34].

### Abiotic stress treatments of peanut plants

For the treatment of polyethylene glycol (PEG6000), sodium chloride (NaCl), or lithium chloride (LiCl), three-leaf-stage (10–15 days after planting) plants were removed from the soil mixture carefully to avoid injury, and then hydroponically grown in a solution for 10 h containing 20% (W/V) PEG6000, 250 mmol/L NaCl, 20 mmol/L LiCl, or deionized water as a control, respectively. For all these treatments, plant samples were frozen in liquid nitrogen immediately following the treatments and stored at −80°C until analysis. The entire experiments were biologically repeated at least three times.

### Molecular cloning of *CYP707A* homolog from peanut

Total RNA was isolated from the frozen samples using the modified phenol-chloroform method as previously described [8]. For amplification of specific homologs encoding CYP707A from peanut, two degenerate primers (DP-707F, 5'-GGN TRI CCI TGY GTI ATG-3'; DP-707R, 5'-ACY TTC CAN CCY TTI GGI AT-3') were designed based on the conserved regions of the reported CYP707As in database. The first-strand cDNA was synthesized from 2 µg of total RNA using the PrimeScript™ II 1st Strand cDNA Synthesis Kit (TaKaRa, Dalian, China) according to the manufacturer's protocol. The cDNA was then used as template for the polymerase chain reaction (PCR) amplification using the degenerate primers DP-707F and DP-707R. The resulting PCR fragments were subcloned, sequenced and compared with the reported *CYP707A* sequences in database. The missing 5' and 3' ends of the amplified fragments were obtained by rapid amplification of cDNA ends (RACE) using the GeneRacer kit according to the manufacturer's instructions (Invitrogen). The gene specific primers for 5' RACE of *AhCYP707A1* were 5GSP-R1-1 (5'-TGC TTT CCC AAC ATC CTC-3') (outer) and 5GSP-R1-2 (5'-TTG TGC TTT GTT CAG CAC-3') (inner); the gene-specific primers for 5' RACE of *AhCYP707A2* were 5GSP-R2-1 (5'-ATG GTA GTG ACC TTG GTG G -3') (outer) and 5GSP-R2-2 (5'-TGT CAC TAA CAC CAA CCG C-3') (inner). The gene specific primers for 3' RACE of *AhCYP707A1* were 3GSP-F1-1 (5'-GAA GAT ACA AAG AAG ATG CC-3') (outer) and 3GSP-F1-2 (5'-GAT GTT GAG TAT CAA GGG-3') (inner); the gene-specific primers for 3' RACE of *AhCYP707A2* were 3GSP-F2-1 (5'-TGC CAT TTA CTC ATA GGG TG-3') (outer) and 3GSP-F2-2 (5'-TTA CAT TTA GGG AGG CTG-3') (inner). In all cloning experiments, PCR fragments were gel-purified with an Agarose Gel DNA Purification Kit (TaKaRa, Dalian, China) and were ligated into the pMD 19-T Vector (TaKaRa, Dalian, China). Plasmids harboring target fragments were isolated and were sequenced from both strands.

### Sequence analyses and alignments

The routine sequence analysis was performed by using Gene Runner (Hastings Software, Inc., New York, NY, USA). Computer analysis of the DNA and amino acid sequences was carried out using the Basic Local Alignment Search Tool (BLAST) program at the National Center for Biotechnology Information Services [35]. Multiple alignments of the amino acid sequences of CYP707As were performed using the Clustal W program in BioEdit software (Isis Pharmaceuticals, Inc., Carlsbad, CA, USA). The full-length CYP707A protein sequences were phylogenetically analyzed by using MEGA 4 software [36] with a bootstrapping set of 1000 replicates. 3D comparative protein structure models of peanut CYP707As were generated with the automatic modeling mode of SWISS-MODEL implemented on the SWISS-MODEL Workspace website (http://swissmodel.expasy.org/) [37], [38]. The protein structures were color-coded. The prediction of the subcellular localization of peanut CYP707A proteins was performed by using the iPSORT algorithm [38] at the website: http://ipsort.hgc.jp/.

### Real-time quantitative RT-PCR performance

The isolated RNA by using the above mentioned method was treated with RNase-free DNase I (TaKaRa, Dalian, China) at 37°C for 1 h to eliminate DNA contamination in real-time quantitative RT-PCR analysis. Two micrograms of total RNA and 200 ng of a random primer were used in reverse transcription (RT) through the cDNA synthesis kit (TaKaRa, Dalian, China) according to the manufacturer's protocol. To investigate the expressions of *AhCYP707A1* and *AhCYP707A2* genes in peanut plants in response to abiotic stresses, the gene-specific primers, GSP-1F (5'-CCT TAA ATG GGT TGC C-3') and GSP-1R (5'-TCA TCA CAA GAC TTC CCC-3') for *AhCYP707A1*, GSP-2F (5'-TTA TTC CTT GCC ACC AGG-3') and GSP-2R (5'-ATT TGT ACT ACT ACC GC-3') for *AhCYP707A2*, were designed to amplify a 252 base pairs (bp), or a 207 bp fragment corresponding to the 3' untranslated region of *AhCYP707A1* or *AhCYP707A2* cDNA for real-time quantitative PCR. The primers 3-UTR-F (5'-GGC TTG ATT ATT TTC CGT TGA GAC CTT TC-3') and 3-UTR-R (5'-CCT GTG CTG GAC AGA AAT TTG CGC AAT ATC-3'), were used to amplify a 433 bp fragment corresponding to the 3' untranslated region of *AhNCED1* cDNA (GenBank accession no. AJ574819), to investigate the expression of *AhNCED1* gene in peanut plants in response to abiotic stresses. As an internal control for normalization of target gene expression, the primers 18S-F (5′-ATT CCT AGT AAG CGC GAG TCA TCA G-3′) and 18S-R (5′-CAA TGA TCC TTC CGC AGG TTC AC-3′) specific to peanut 18S rRNA gene (GenBank accession no. AF156675) were used to amplify a fragment of 226 bp. Real-time quantitative PCRs were performed in the presence of Power SYBR green PCR Master Mix (Applied Biosystems, Guangzhou, China). Amplification was monitored in real-time with the MiniOpticon™ Real-Time PCR System (Bio-Rad, Shanghai, China). The products of real-time quantitative PCR were confirmed by determining the melt curves for the products at the end of each run, by analysis of the products using gel electrophoresis, and by sequencing. Quantification of the normalized gene expression was performed with the comparative cycle threshold (Ct) method [40]. Three biological and three technical replicates were performed for each experiment. All RT-PCR data were expressed as the mean ± standard error. Statistical differences of expressions of *AhCYP707A1* and *AhCYP707A2* among peanut organs or various treatments were assessed by one-way analysis of variance (ANOVA) followed by the least significant difference (LSD) and Student-Neumann-Keuls (SNK) *post hoc* comparison. The analyses were performed with SPSS 13.0 software (SPSS Inc., Chicago, IL, USA). The threshold of significance was defined as *p*<0.05.

### Functional expression of peanut *CYP707A1* in yeast

The open reading frame of *AhCYP707A1* cDNA was amplified by standard PCR. The resulting product was sequenced to confirm the absence of PCR-caused mutation, and was cloned into a yeast expression vector, pYESDEST52 [16], [41]. The resulting plasmid was transformed into *Saccharomyces cerevisiae* strain WAT11 [41]. Transformants were grown in SGI medium (3.4 g/L yeast nitrogen base, 5 g/L bactocasamino acid, 20 g/L glucose and 40 mg/L tryptophan) for 24–36 h, transferred to SLI medium, which is identical to SGI, except that glucose is replaced by 20 g/L galactose, for induction by galactose for 12 h [11]. To prepare microsomal proteins, yeast cells collected from a 500-ml SLI culture were suspended in 0.1 mol/L potassium phosphate buffer (pH 7.6). Disruption and subcellular fractionation of yeast cells were performed with a modified procedure that combined mechanical rupture and enzymatic lysis methods to improve fractionation efficiency [42]. The broken cells were centrifuged at 10 000 g for 15 min, and the supernatant was centrifuged at 140 000 g for 1 h. The pellet was washed briefly with 0.1 mol/L potassium phosphate (pH 7.6), and the microsomal fraction was resuspended in the same buffer plus 5% glycerol. Protein was quantified using a Bio-Rad protein assay kit (Bio-Rad, Hercules, CA, USA). The ABA 8'-hydroxylase enzymatic assay contained 100 µg of microsomal protein in 0.1 mL of 50 mmol/L potassium phosphate (pH 7.6) to which was added 3 µL of 10 mmol/L ABA and 1 µL of 10 mmol/L NADPH. After incubation at 22°C for 18 h, the reaction was stopped by addition of 10 µL acetic acid. The reaction products were extracted four times with an equal volume of ethyl acetate. The ethyl acetate extracts were resuspended in 100 µL of methanol, and 5 µL of the sample was subjected to HPLC analysis on the Sep-Pak C18 cartridges (Waters, Milford, MA, USA) with a 40-min gradient from 10% to 80% methanol in water containing 0.1% acetic acid and a flow rate of 1 mL/min (UV detection at 254 nm). The retention time for authentic PA and ABA was 16.9 and 36.8 min, respectively.

### Determination of ABA level

The extraction and quantification of endogenous ABA were performed as previously described [9], [31]. The ABA levels were determined from three independent experiments with three replicates for each.

## Results

### Cloning and characterization of genes AhCYP707A1 and AhCYP707A2 encoding ABA 8'-hydroxylase in peanut plants

The conserved regions of the reported plant CYP707As were used for the design of degenerate primers used in the PCR amplification of new *CYP707A* homolog from peanut plants. Two fragments (named as *AhCYP707A1* and *AhCYP707A2*, respectively) were amplified from the cDNA of PEG6000-treated peanut leaves. The sequences of the two fragments show high similarity with the reported *CYP707A*s in GenBank DNA database. The full length cDNA of *AhCYP707A1* obtained through RACE, consists of 1730 bp nucleotides, including a 91-bp 5' untranslated region (5' UTR) and a 238-bp 3' untranslated region (3' UTR). *AhCYP707A1* cDNA has an open reading frame (ORF), encoding a polypeptide of 466 amino acid residues with a calculated molecular weight of 53.39 kDa and an isoelectric point of 9.16. The full length cDNA of *AhCYP707A2* obtained through RACE, consists of 2246 bp nucleotides, including a 443-bp 5' UTR and a 357-bp 3' UTR. *AhCYP707A2* cDNA has an ORF encoding a polypeptide of 481 amino acid residues with a calculated molecular weight of 55.06 kDa and an isoelectric point of 9.22.

**Figure 1:**
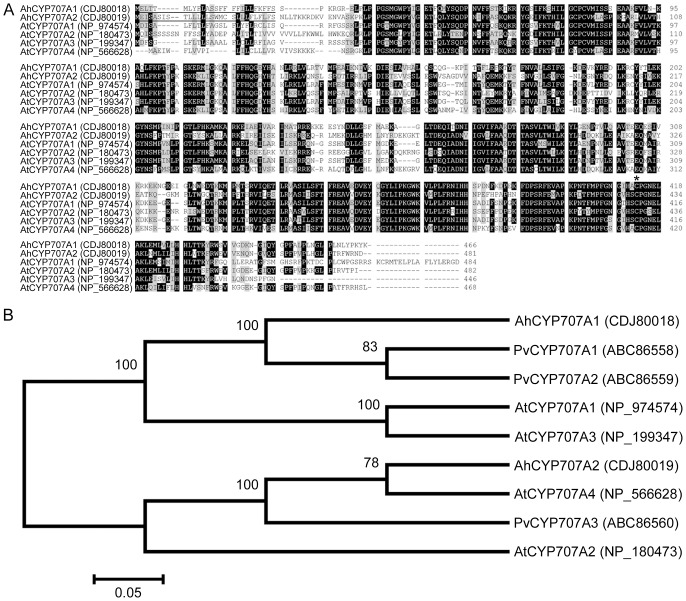
Sequence analyses of CYP707A proteins from peanut, Arabidopsis and bean. (A) Alignment of deduced amino acid sequences of peanut AhCYP707A1, AhCYP707A2 with four Arabidopsis CYP707A proteins (AtCYP707A1 to 4). Identical and similar amino acid residues were shaded in black and gray, respectively. The asterisk symbol above the alignment indicates the active-site cysteine of CYP707As. A putative signal peptide located at the N-terminus of AhCYP707A1 or AhCYP707A2 was underlined. Amino acids were numbered from the initial methionine. GenBank accession numbers for each aligned CYP707A protein were indicated in parentheses. (B) Phylogenetic analysis of amino acid sequences of AhCYP707A1, AhCYP707A2 with four Arabidopsis CYP707As (AtCYP707A1 to 4) and three bean CYP707As (PvCYP707A1 to 3). Multiple sequence alignment was performed using Clustal W and the phylogenetic tree was constructed via the Neighbor-Joining method in MEGA 4 software. Bootstrap values from 1000 replicates for each branch were shown. GenBank accession numbers for each analyzed CYP707A were indicated in parentheses. The scale bar is 0.05.

**Figure 2:**
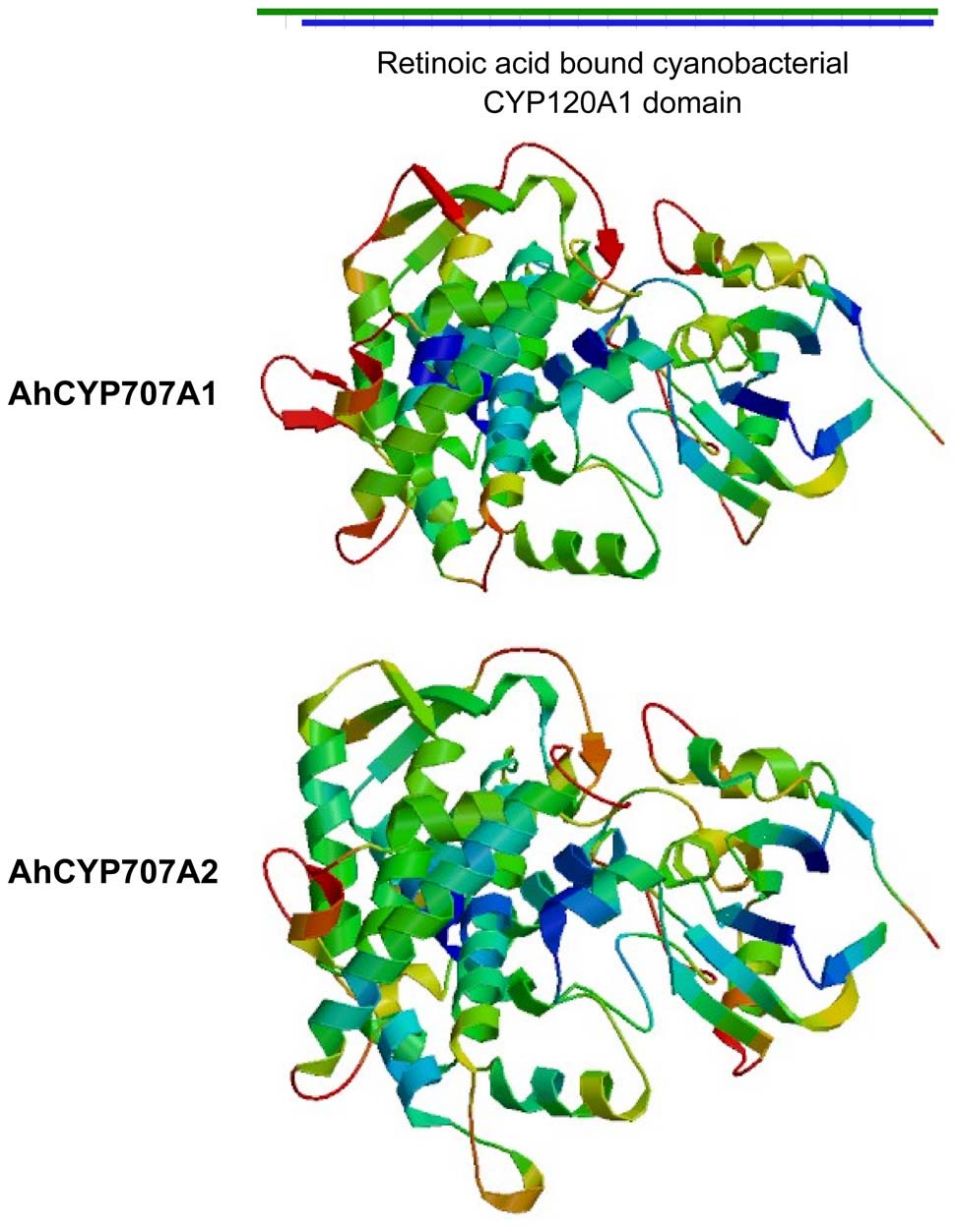
Ribbon diagrams of major 3D structure of AhCYP707A1 and AhCYP707A2 generated with SWISS-MODEL. The green bar above the ribbon diagrams represents protein sequences with their corresponding amino acid lengths, and the blue bar indicates the sequence areas where 3D structure was generated with SWISS-MODEL. The structure was color-coded ranging from the *N*-terminus (blue) to the *C*-terminus (red).

Multiple sequence alignments (Figure 1A) of the deduced amino acids of AhCYP707A1 and AhCYP707A2 showed that AhCYP707A1 and AhCYP707A2 shared 51.9% sequence identity with each other. AhCYP707A1 shared 67.8%, 67.0%, 55.4% and 51.8% sequence identity with Arabidopsis AtCYP707A3, AtCYP707A1, AtCYP707A2, and AtCYP707A4, respectively; AhCYP707A2 shared 67.6%, 53.0%, 52.1% and 51.0% sequence identity with Arabidopsis AtCYP707A4, AtCYP707A2, AtCYP707A3, and AtCYP707A1, respectively (Figure 1A). Both AhCYP707A1 and AhCYP707A2 proteins contain the highly conserved cysteine residue (PFGNGTHSCPG), which is the putative heme iron ligand, and appears to be essential for catalysis [11]. Both AhCYP707A1 and AhCYP707A2 are predicted as having a signal peptide by the iPSORT prediction (Figure 1A) [39]. Phylogenetic analysis of AhCYP707A1 and AhCYP707A2, four Arabidopsis CYP707As and three bean CYP707As showed that AhCYP707A1 protein was closer to bean PvCYP707A1 and PvCYP707A2, Arabidopsis AtCYP707A1 and AtCYP707A3, and that AhCYP707A2 protein was closer to Arabidopsis AtCYP707A4 (Figure 1B), which have all been proved actively in ABA 8'-hydroxylation [11], [16]. The SWISS-MODEL tool generated a 3D structure for the domain of retinoic acid bound cyanobacterial CYP120A1 in both AhCYP707A1 and AhCYP707A2 proteins (Figure 2). These results imply that both of peanut CYP707As likely function as an active ABA 8'-hydroxylase homologous to other characterized CYP707A proteins.

The complete cDNA sequences of *AhCYP707A1* and *AhCYP707A2* genes have been deposited in the EMBL nucleotide sequence database under the accession numbers HG764750 and HG764751, respectively.

### Organ specific expression patterns of *AhCYP707A1* and *AhCYP707A2* in peanut seedling

**Figure 3:**
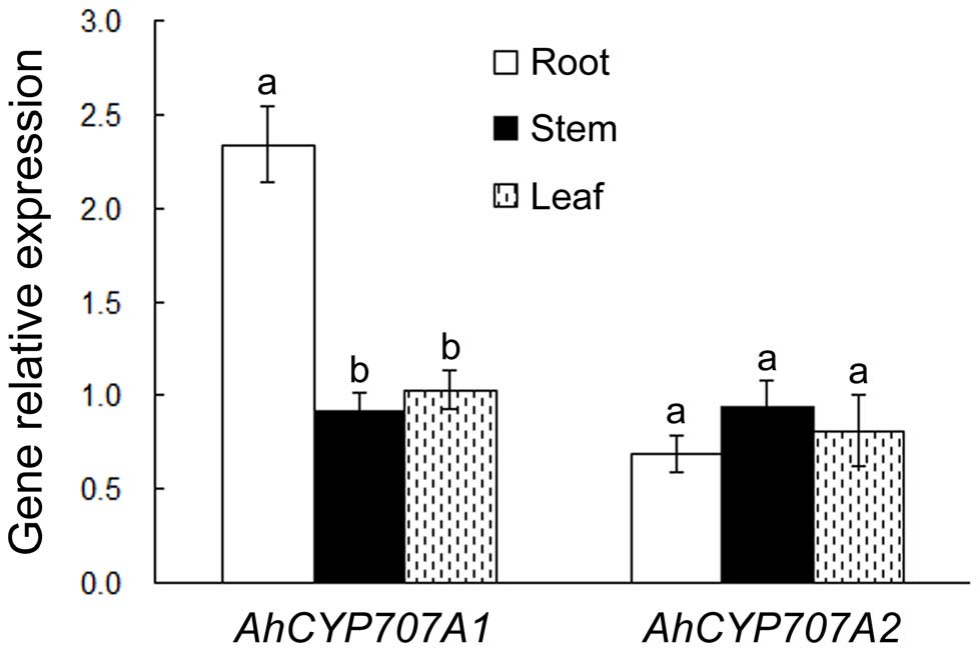
Expressions of *AhCYP707A1* and *AhCYP707A2* in peanut under normal conditions were determined by real-time RT-PCR. Total RNA was prepared respectively from root, stem, and leaf of peanut plants. Gene expressions were shown relative to the expression of peanut 18S rRNA gene in each sample. All data are presented as mean ± standard errors (SE) of three replicates. A different letter above each bar indicates a significant difference between organs (*p*<0.05).

Real-time quantitative RT-PCR analysis was performed to examine the expressions of *AhCYP707A1* and *AhCYP707A2* in 14-day-old peanut seedlings (Figure 3). *AhCYP707A1* and *AhCYP707A2* genes were expressed in all organs examined, including roots, stems and leaves, although the relative abundance differed between genes. In particular, *AhCYP707A1* mRNA predominantly accumulated in roots. The *AhCYP707A2* gene was ubiquitously expressed in roots, stems and leaves of peanut.

### Transcripts of AhCYP707A1, AhCYP707A2 and AhNCED1 and ABA levels in peanut seedling were up-regulated by osmotic stress but not by ionic stress

**Figure 4:**
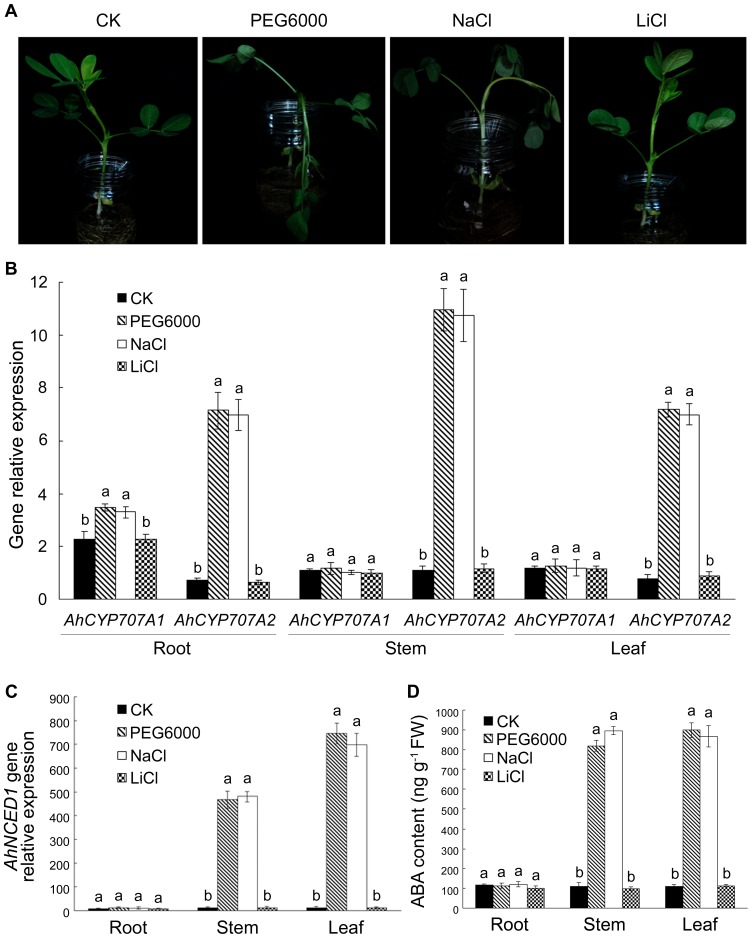
Effects of osmotic and salt stresses on peanut seedlings. Peanut seedlings of twelve days old were hydroponically grown in the solution containing 20% PEG6000, 250 mmol/L NaCl, 30 mmol/L LiCl, or deionized water as a control (CK) for 10 h to assess wilting symptoms visually. Photographs shown here represent the results of triplicate independent assays (A). Organ specificity of the accumulation of *AhCYP707A1*, *AhCYP707A2* (B) and *AhNCED1* (C) transcripts, and endogenous ABA (D) in peanut plants in response to PEG6000, NaCl or LiCl. Total RNA was prepared separately from root, stem and leaf of stressed or control (CK) plants. Real-time quantitative RT-PCR analysis was performed as described in Figure 3. The ABA levels in the root, stem, and leaf of peanut plants at the presence or absence of stresses were measured triplicately for each sample. All data are presented as mean ± SE of three replicates. A different letter above each bar indicates a significant difference between treatments (*p*<0.01).

The expressions of four Arabidopsis *AtCYP707A*s [11], [12], rice *OsCYP707A5* [15] and ten soybean *GmCYP707A*s [18] were reported to be induced by both dehydration and high salinity. In the present study, the expressions of *AhCYP707A1*, *AhCYP707A2* and *AhNCED1* genes in peanut plants in response to 20% PEG6000 or 250 mmol/L NaCl were determined by real-time quantitative RT-PCR performance, and the endogenous ABA levels were simultaneouly determined. As shown in Figure 4B, the expression of *AhCYP707A1* gene was up-regulated by PEG6000 or NaCl in peanut roots, but not in stems and leaves. By contrast, the transcript levels of *AhCYP707A2* in roots, stems and leaves were all increased significantly by those treatments. The expression of *AhNCED1* gene was strongly up-regulated by PEG6000 or NaCl in leaves and stems, but not in roots (Figure 4C). The endogenous ABA also accumulated predominantly in leaves and stems under those treatments (Figure 4D). To examine whether the effect of NaCl might be related to osmotic or ionic stress, we examined the effects of 30 mmol/L LiCl, at which concentration osmotic status of cells is not seriously affected, the toxicity of Li^+^ being even higher than that of Na^+^ ions [25]. The growth of peanut plants was evaluated visually. After a 10-h treatment, peanut seedlings hydroponically grown in the solution containing 20% PEG6000 or 250 mmol/L NaCl showed severe wilting symptoms, whereas no significant wilting symptom was observed in plants grown in the solution containing 30 mmol/L LiCl, suggesting the susceptibility to osmotic stress and the resistance to salt ions in peanut seedlings (Figure 4A). The effect of 30 mmol/L LiCl on the levels of *AhCYP707A1*, *AhCYP707A2* and *AhNCED1* transcripts and endogenous ABA in peanut plants was further determined. The result showed that the mRNA levels of *AhCYP707A1*, *AhCYP707A2* (Figure 4B) and *AhNCED1* (Figure 4C) were not affected by the LiCl treatment. The ABA level was also not affected by the LiCl treatment (Figure 4D). The significant up-regulation of *AhCYP707A1*, *AhCYP707A2* and *AhNCED1* transcripts and endogenous ABA levels by both 20% PEG6000 and 250 mmol/L NaCl, but not by 30 mmol/L LiCl, showed that the osmotic stress instead of ionic stress affected the expression of those genes and the biosynthesis of ABA in peanut plants.

### ABA 8'-hydroxylase activity of recombinant AhCYP707A1 protein

**Figure 5:**
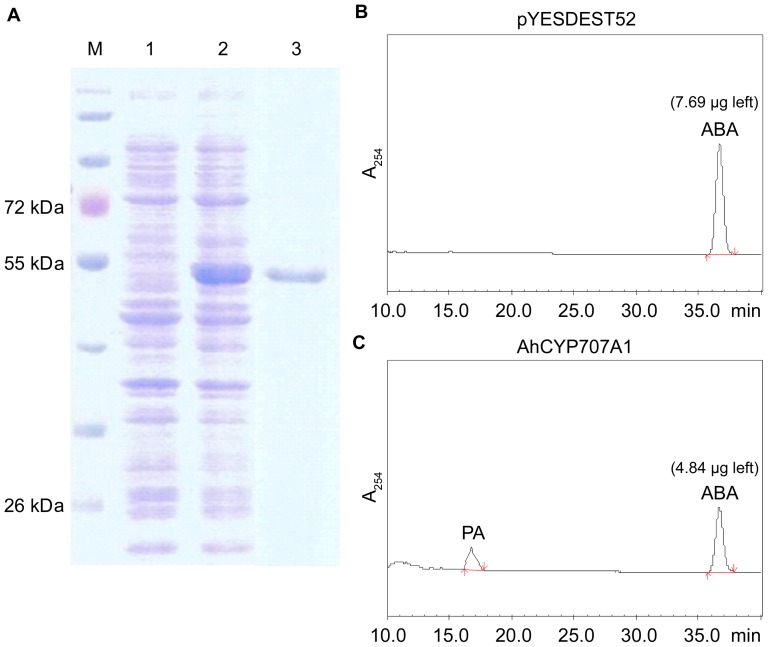
Functional expression of AhCYP707A1 in yeast. (A) SDS-PAGE of microsomes isolated from yeast cells expressing the plasmid pYESDEST52 only (1), or expressing recombinant AhCYP707A1 (2). Lane 3 is the purified recombinant AhCYP707A1. (B) HPLC analysis of reaction products of ABA (7.69 µg left) incubated with microsomes from yeast cells expressing the plasmid pYESDEST52 only. (C) HPLC analysis of reaction products of ABA (4.84 µg left) incubated with microsomes from yeast cells expressing AhCYP707A1. The recombinant AhCYP707A1 microsomes (100 µg protein) were incubated with ABA (7.93 µg). Retention time is given in minutes, while the vertical axis indicates UV absorbance at 254 nm (A_254_). The positions of ABA and PA are indicated.

The recombinant AhCYP707A1 protein was expressed in yeast [41] to test for the enzymatic activity of converting ABA to PA. SDS-PAGE analysis showed that a new intense band of about 53 kDa appeared in the microsomal fraction of yeast cell expressing AhCYP707A1 (Figure 5A). As an *in vitro* test, microsomes isolated from yeast cell expressing AhCYP707A1 protein was analyzed for the function of ABA degradation. HPLC analyses revealed that the ABA level (4.84 µg) [the peak at the retention time of 36.8 min (Figure 5C)] was greatly decreased in the microsomes isolated from yeast cell expressing AhCYP707A1, whereas the ABA level (7.69 µg) remained nearly unchanged in the microsomes isolated from yeast with the plasmid only (Figure 5B). Furthermore, one new peak was detected in the same analysis at the retention time of 16.9 min, whereas the microsomes isolated from yeast with the plasmid only produced no new peak at that retention time (Figure 5B and C). The new peak was identical in the retention time to authentic standard of PA.

## Discussion

Regulation of ABA metabolism within plants depends on internal or external signals, such as developmental stages or water deficit. The equilibration of ABA biosynthesis and catabolism is a determinant of endogenous ABA content in plants. ABA is catabolized into an inactive form either by oxidation or conjugation, and it has been generally assumed that ABA 8'-hydroxylation, catalyzed by the CYP707A proteins, plays a prominent role in ABA catabolism [11], [12]. The CYP707A family appears to be highly conserved among different plant species. In the present study, two cDNAs, *AhCYP707A1* and *AhCYP707A 2*, encoding ABA 8'-hydroxylase were cloned and characterized from peanut plants. Multiple sequence alignments showed that both peanut CYP707As share high similarity with previously reported CYP707As in database (Figure 1). The active site of cysteine residue [11] was well conserved in both peanut CYP707As (Figure 1A). In a comparison of deduced amino acid sequences, AhCYP707A2 shares highest sequence identity with AtCYP707A4, which was consistent with the result that AhCYP707A2 is phylogenetically closer to AtCYP707A4 (Figure 1B). AhCYP707A1 and AhCYP707A2 are both predicted as having a signal peptide (Figure 1A). The 3D structure for the domain of retinoic acid bound cyanobacterial CYP120A1 was generated in both AhCYP707A1 and AhCYP707A2 proteins (Figure 2). The function of the AhCYP707A1 was demonstrated by heterologous expression in yeast (Figure 5). The microsomal fraction prepared from yeast cell expressing AhCYP707A1 produced, in addition to an ABA peak, a PA peak in the HPLC analysis, whereas the control expressing the plasmid only did not produce a PA peak (Figure 5B and C). These results from *in vitro* assay conclusively demonstrate that the peanut AhCYP707A1 protein exhibits the catalytic activity of ABA 8'-hydroxylase.

In Arabidopsis, *CYP707A* genes were expressed in all tissues, although the relative abundance differed among genes. Overall expression levels of *CYP707A1* and *CYP707A3* are much higher than those of *CYP707A2* and *CYP707A4*; flower buds and flowers were relatively abundant in the transcripts of all *CYP707A*s compared with the other tissues; in rosette leaves, *CYP707A3* mRNA was the major transcript, while *CYP707A2* was abundantly expressed in inflorescence stems; in roots, *CYP707A1* and *CYP707A3* were moderately expressed, whereas weak expression of *CYP707A2* and no expression of *CYP707A4* were observed [12]. Transcripts of three bean *PvCYP707A*s were relatively abundant in mature organs, stems and roots, whereas young organs showed low expression levels of *PvCYP707A*s [16]. In rice, *CYP707A5* was highly expressed in seedlings and expanding leaves, though *CYP707A6* was highly expressed in internodes and expanding leaves, but showed weak expression in seedlings [15]. Here we showed that peanut *AhCYP707A1* and *AhCYP707A2* genes were expressed ubiquitously in roots, stems and leaves with different transcript accumulation levels, including the higher expression of *AhCYP707A1* in roots (Figure 3). These results indicate that the expression of plant *CYP707A* genes is differently regulated in each organ, although there is functional redundancy among the *CYP707A* genes in plants [11], [12], [15], [16], [18].

It has been shown that the level of ABA increases dramatically in response to osmotic stress [43]. The accumulation of endogenous ABA that occurs under osmotic stress is rapidly reversed through ABA catabolism subsequent to removal of the stress [20], [44]. It is important to note that these reactions occur concomitantly with the induction of *CYP707A*s gene expression [11], [12], [16]. Saito et al [12] showed that the transcript levels of all four Arabidopsis *CYP707A* genes increased in response to mannitol or drought stress. Water stress strongly induced the expression of *PvCYP707A3* instead of *PvCYP707A1* and *PvCYP707A2* in bean leaves [16]. In rice, *CYP707A5* showed sharp increases of transcripts in mannitol-treated leaves, whereas *CYP707A6* did not respond to the treatment [15]. In this study, we demonstrated that the transcript levels of both peanut *CYP707A* genes increased in response to PEG6000- or NaCl-induced osmotic stress (Figure 4B). The expression of *AhCYP707A2* gene was significantly up-regulated by PEG6000 or NaCl in peanut roots, stems and leaves, whereas the up-regulation of *AhCYP707A1* mRNA level by PEG6000 or NaCl was not observed in stems and leaves except for roots (Figure 4B), implying that AhCYP707A1 might be involved in ABA catabolism in peanut roots in response to osmotic stress.

The expression of *CYP707A* genes induced by high concentration of NaCl has been observed in Arabidopsis [12], rice [15], and soybean [18]. In the present study, the expressions of *AhCYP707A2* in peanut roots, stems and leaves, and *AhCYP707A1* in roots were sharply induced by 250 mmol/L NaCl (Figure 4B). Since NaCl has both osmotic and ionic effects simultaneously [24], low concentration of LiCl was used to examine whether the effect of NaCl might be related to osmotic or ionic stress. As shown in Figure 4, the significant up-regulation of *AhCYP707A1*, *AhCYP707A2* and *AhNCED1* transcripts and endogenous ABA levels by both PEG6000 and NaCl, but not by LiCl, showed that the osmotic stress instead of ionic stress affected the expression of those genes and the biosynthesis of ABA in peanut plants. The susceptibility to osmotic stress and the resistance to salt ions were observed in peanut seedlings.

Umezawa et al [21] suggested that CYP707A3 is the major enzyme for ABA catabolism in Arabidopsis during dehydration, whereas AtNCED3 has a significant role in dehydration-responsive ABA biosynthesis [7]. We have previously shown that the expression of *AhNCED1* gene in peanut plants is significantly up-regulated by dehydration, and suggested that the expression of *AhNCED1* gene plays an important role in water-stress induced biosynthesis of ABA [8], [9]. ABA levels are maintained by the balance between its biosynthesis and catabolism, rather than simply by the biosynthesis alone. Our results demonstrated that the expression of both peanut *CYP707A* genes was activated upon osmotic stress (Figure 4B), although their induction was much slighter than that of *AhNCED1* expression (Figure 4C). This difference in induction kinetics may define the accumulation of stress-induced ABA levels (Figure 4D). In response to osmotic stress, the balance between ABA biosynthesis and catabolism might be explained as follows. It is likely that stress-responsive induction of CYP707As helps not only to maintain endogenous ABA levels within the permissible range, but also to prepare the plant for degradation of ABA after removal of the stress. It is proposed that ABA- biosynthetic and catabolic activities co-operatively determine endogenous ABA levels in plants during osmotic stress.

In conclusion, the present work has identified two genes, *AhCYP707A1* and *AhCYP707A2* encoding the key enzyme in ABA catabolism, ABA 8'-hydroxylase, in peanut. The heterologous expression in yeast demonstrated the catalytic activity of ABA 8'-hydroxylase of the recombinant AhCYP707A1 protein. Expressions of *AhCYP707A1* and *AhCYP707A2* genes in peanut plants were regulated osmotically, as shown by responses to osmotic stress instead of ionic stress. Our present findings demonstrate that the expressions of *AhCYP707A1* and *AhCYP707A2* play a vital role in ABA catabolism in peanut, particularly in response to osmotic stress. Further analyses will be required to understand the *in planta* functions of both peanut *CYP707A* genes, and these studies will help to discriminate the possible functional redundancy and differences among *CYP707A* gene family.
